# Diagnostic Discrepancies Between Emergency Department Assessment and Internal Medicine Discharge Diagnosis: A Prospective Observational Study in a Swiss Tertiary Hospital

**DOI:** 10.3390/jcm15187177

**Published:** 2026-09-15

**Authors:** Theresa Ackfeld, Youcef Guechi, Joseph Schwab, Thomas Castelain, Ludovic Galofaro, Cynthia Gay, Sébastien Pugnale, Thomas Schmutz, Wolf E. Hautz, Vincent Ribordy

**Affiliations:** 1 Department of Emergency Medicine, HFR-Cantonal Hospital, 1700 Fribourg, Switzerland; youcef.guechi@h-fr.ch (Y.G.); thomas.castelain@h-fr.ch (T.C.); galofaro.ludovic@gmail.com (L.G.); cynthiagay3@gmail.com (C.G.); sebastien.pugnale@h-fr.ch (S.P.); vincent.ribordy@h-fr.ch (V.R.); 2 Faculty of Science and Medicine, Section of Medicine, University of Fribourg, 1700 Fribourg, Switzerland; 3 Education and Health Promotion Laboratory, Sorbonne Paris Nord University, 93430 Villetaneuse, France; 4 Department of Orthopedic Surgery and Traumatology, HFR-Cantonal Hospital, 1700 Fribourg, Switzerland; joseph.schwab@unifr.ch; 5 Department of Emergency Medicine, EHC-Coast Hospital Group, 1110 Morges, Switzerland; thomas.schmutz@ehc.vd.ch; 6 Department of Emergency Medicine, Inselspital University Hospital, University of Bern, 3010 Bern, Switzerland; wolf.hautz@insel.ch

**Keywords:** diagnostic error, diagnostic discrepancy, emergency department, quality in health care, diagnostic performance, patient safety

## Abstract

**Background/Objective**: Diagnostic safety is a major challenge in emergency departments (ED), where clinicians frequently make decisions under time pressure and with incomplete information. Prospective data on diagnostic discrepancies in Swiss EDs remain limited. To determine the frequency of diagnostic discrepancies between the initial ED diagnosis and the final Internal Medicine (IM) discharge diagnosis, identify associated patient-, physician-, and context-related factors, and evaluate clinical outcomes associated with diagnostic discrepancy. **Methods**: We conducted a prospective observational study including 515 patients admitted from the ED to an IM ward and managed by 44 physicians at a Swiss tertiary non-university hospital. The initial ED diagnosis was compared with the IM discharge diagnosis (or diagnosis on day 28 if the patient remained hospitalized). Diagnostic discrepancies were classified using a predefined algorithm with independent expert opinion where required. Generalized linear mixed-effects models were used to assess associations between diagnostic discrepancy and mortality, in-hospital transfers and length of stay; a multivariable logistic regression model was used to identify factors associated with diagnostic discrepancy. **Results**: Diagnostic discrepancies were identified in 10.1% of patients (*n* = 52). These patients had longer hospital stays (9.3 ± 9.2 vs. 6.9 ± 5.8 days; *p* = 0.069) and were more frequently transferred within the hospital (17% vs. 5.8%; *p* = 0.006). After adjustment, diagnostic discrepancies were associated with higher odds of in-hospital transfer (OR 3.35; 95% CI 1.47–7.66; *p* = 0.004) and a 20% longer stay (exp β = 1.20; 95% CI 1.00–1.45; *p* = 0.049), although the latter was attenuated after adjustment for comorbidity. Specialist involvement in the ED was independently associated with lower odds of diagnostic discrepancy (OR 0.30; 95% CI 0.10–0.72; *p* = 0.015), whereas each one-point increase in physician-perceived diagnostic difficulty was associated with higher odds (OR 1.42; 95% CI 1.07–1.90; *p* = 0.016). **Conclusions**: Diagnostic discrepancies occurred in approximately one in ten patients admitted from the ED to IM and were associated with increased in-hospital transfer and, less robustly, with prolonged hospitalization. Prospective multicenter studies should evaluate strategies to reduce diagnostic discrepancies and improve diagnostic safety.

## 1. Introduction

### 1.1. Context

Diagnostic error is an important concern for patient safety [1]. It is associated with mortality and morbidity [2] and with increased length of hospital stay [3,4], and it accounts for 6–17% of hospital adverse events [5]. Diagnostic errors are the leading cause of paid medical malpractice claims [5,6], lead to delays in adequate therapy and therefore contribute substantially to the economic burden on the healthcare system [5]. A recent report from the Organization for Economic Co-operation and Development (OECD) estimated that more than 17% of healthcare costs in an average OECD country may be attributable to suboptimal diagnostic processes [7].

Emergency departments (EDs) are particularly vulnerable to diagnostic error because clinical decisions must often be made under considerable time pressure, with incomplete information and while treating an increasing number of patients at the same time. Furthermore, overcrowding has become a substantial problem. Consequently, diagnostic performance in the ED has become an important area of patient safety research.

Most of this evidence is framed around the concept of diagnostic error, but because this term is difficult to operationalize, we specifically study diagnostic discrepancies, a related but distinct and more directly measurable construct (see Diagnostic Error versus Diagnostic Discrepancy). Internationally, diagnostic error rates in the ED vary between 5.7% and 50% and were even found to be as high as 64% for patients presenting with non-specific complaints [1,8,9]. Most studies estimate the ED error rate to be between 10% and 15% [10].

The most recent prospective study from Switzerland showed that one in nine to ten diagnoses might be wrong [4]. By contrast, a meta-analysis from the US, considering mostly retrospective analyses, showed a diagnostic error in every 18 patients, a diagnostic adverse event in every 50 patients, serious harm (serious disability or death) in about every 350 patients, and a misdiagnosis-related death in about every 500 patients [1].

Several studies have examined predictors of diagnostic errors, with mixed results. Earlier work linked patient demographics, chief complaint, time pressure, and physician overconfidence to diagnostic changes, but a recent Swiss study found no such associations [4,5,8,9,11,12,13,14,15].

While diagnostic errors frequently involve multiple contributing factors, a review showed that flawed clinical decision-making (errors in assessment, test ordering, and interpretation) or judgment was the predominant cause in 89% (95% CI 88–90) of malpractice claims and that most often these were attributed to inadequate knowledge, skills, or reasoning [1].

### 1.2. Rationale

Over the past two decades, increasing attention has been paid to the incidence, contributing factors and consequences of diagnostic errors [16]. In European hospitals, diagnostic errors continue to represent a blind spot in patient safety, imposing a significant burden on both patients and healthcare providers.

Most studies investigating diagnostic errors rely on retrospective analyses, which are susceptible to documentation and selection biases. Additional limitations include heterogeneous definitions of diagnostic error, selection bias, differing time windows for outcome assessment, and incomplete outcome ascertainment due to missing follow-up data [1,10,17].

Diagnostic errors are difficult to detect because of heterogeneous definitions, evolving disease processes, and the influence of hindsight bias when retrospectively assessing whether a diagnosis should have been established at the time of the initial presentation. Furthermore, the inclusion of both underdiagnosis and overdiagnosis within the concept of diagnostic error complicates its evaluation [17,18]. Recent data from Switzerland have improved our understanding of diagnostic performance in the ED [4] and warrant further research, especially in other Swiss institutions.

### 1.3. Diagnostic Error Versus Diagnostic Discrepancy

The definition of diagnostic error varies considerably in the literature. For the purpose of this study, we defined diagnostic error, according to the definition of Graber et al. as a “diagnosis that was unintentionally delayed […], wrong […], or missed […], as judged from the eventual appreciation of more definitive information” [4,16].

Because diagnostic error is difficult to measure directly, many studies have evaluated diagnostic discrepancies or diagnostic changes between the initial ED diagnosis and a later diagnosis established during hospitalization. Given the challenges in defining and measuring diagnostic error, as well as the judgmental connotation of the term, we here assessed diagnostic discrepancies, defined as a change in diagnosis between the ED admission and internal medicine discharge.

While diagnostic discrepancy and diagnostic change are descriptive and denote a variation between two diagnostic assessments (i.e., a labeling error), without implying that a mistake in the process has occurred, diagnostic error is commonly used as the sum of labeling and process errors.

Although preceding studies showed that the hospital’s discharge diagnosis is not necessarily the correct diagnosis, we here use it as our reference standard [9], while acknowledging that it does not constitute a validated gold standard for diagnostic correctness. Nevertheless, there are several reasons why the hospital’s discharge diagnosis is potentially more accurate than the initial ED diagnosis: patients usually stay several days at the ward, which makes it possible to gather more information (e.g., by calling the patient’s family doctor), spend more time with the patient, have access to further tests and obtain results of time-consuming investigations. Furthermore, interdisciplinary discussion is possible and ward staff can observe patient improvement (or lack thereof) for days, rather than hours in the ED.

For consistency, we use diagnostic error only when referring to findings from the literature that used this term, and diagnostic discrepancy when referring to our own outcome measure throughout this manuscript.

### 1.4. Objective

The aim of this study was to evaluate diagnostic discrepancies between the initial ED diagnosis and the final diagnosis at discharge from the Internal Medicine (IM) ward at the Fribourg Cantonal Hospital (HFR). The specific objectives were to:

1. Determine the frequency of diagnostic discrepancies between the initial ED diagnosis and the final IM discharge diagnosis.
2. Describe outcomes associated with a change in diagnosis, including length of hospital stay, transfer to another hospital unit, and in-hospital mortality.
3. Identify patient-, physician-, and context-related factors associated with a change in diagnosis.

## 2. Materials and Methods

### 2.1. Study Design

We conducted a single-center prospective observational study between July 2023 and November 2024 using documented health-related personal data. No biological or genetic material was obtained.

The Fribourg Cantonal Hospital is a tertiary hospital in Switzerland that receives around 42,000 emergency department patient visits per year. Patients were seen by residents who then discussed each case with a senior physician specializing in internal medicine or emergency medicine. We included patients admitted from the ED to any internal medicine (IM) ward.

We collected physician-, patient-, and context-related factors that were present on admission to the ED. ED diagnosis and IM ward discharge diagnosis (or diagnosis on day 28 if not discharged until then) were compared to assess a change in diagnosis. Whether or not a change in diagnosis occurred was decided based on a dedicated algorithm.

### 2.2. Participants and Eligibility Criteria

All patients aged 16 or older who were hospitalized via the ED to the IM ward at HFR-Fribourg were screened for inclusion over a 16-month period until 500 patients were included. Inclusion criteria were voluntary consent and admission to the IM ward via the ED. Patients were only included if a case-specific questionnaire was completed by the physician in charge. Exclusion criteria were admission to the ICU, orthopedic ward, surgical ward, or palliative care; ambulatory management; missing oral consent; missing physician questionnaire; and admission to a hospital other than Fribourg within the HFR network.

Physicians were included if they agreed to participate and completed a baseline questionnaire. All physicians and sixth-year medical students could participate. Physicians received 10 CHF for each completed patient-specific questionnaire. The trial was registered under: 2Req-023-00651.

This study was conducted in accordance with the Declaration of Helsinki. Ethics committee approval was requested but deemed unnecessary, as this was a quality evaluation study according to Swiss research legislation. In accordance with the Cantonal Ethics Committee for Research Involving Human Subjects (CER-VD), Lausanne, Switzerland, and data protection laws, informed consent was not required for studies of this nature [19].

### 2.3. Data Collection

Data were collected prospectively from three sources:

1. Patient information was imported from the digital patient record Medfolio V (NEXUS Schweiz AG, Schenkon, Switzerland) using a unique patient identifier and included: demographics, comorbidities, frailty, triage category, admission and discharge times, diagnoses, and outcomes (length of stay, mortality, transfers).
2. Physician characteristics were collected using a standardized physician questionnaire that each physician completed at the beginning of the study. This questionnaire gathered essential demographic and professional information, including age, years of experience, primary language, specialty, and board certification status (Supplemental Data S1).
3. Context factors and physician-reported predictors were collected with a questionnaire that was completed by each physician, immediately after the patient had been admitted to IM or on the same day at the end of the shift. The questionnaire contained information such as perceived challenges in establishing the diagnosis, communication barriers with the patient, clinician fatigue, familiarity with the particular diagnosis, and other relevant factors (Supplemental Data S2). Once included, physicians identified eligible patients according to inclusion and exclusion criteria during routine clinical practice in ED. When a patient met the inclusion criteria, oral patient consent was obtained. Patient diagnosis, treatment and data were not influenced by the inclusion into this study. Routine healthcare data necessary for diagnosis and treatment were collected as usual.

Patients were subsequently monitored for a period of 28 days post-admission or until discharge, at which point their diagnoses from the ED and the internal medicine ward were documented in a centralized database (REDCap, Vanderbilt University, Nashville, TN, USA). In addition to diagnostic information, several outcome factors such as mortality, transfers to other facilities, and length of hospitalization were collected for each patient.

Patient and physician anonymity was maintained at all times by anonymizing their data.

### 2.4. Diagnostic Discrepancy

**Figure 1:**
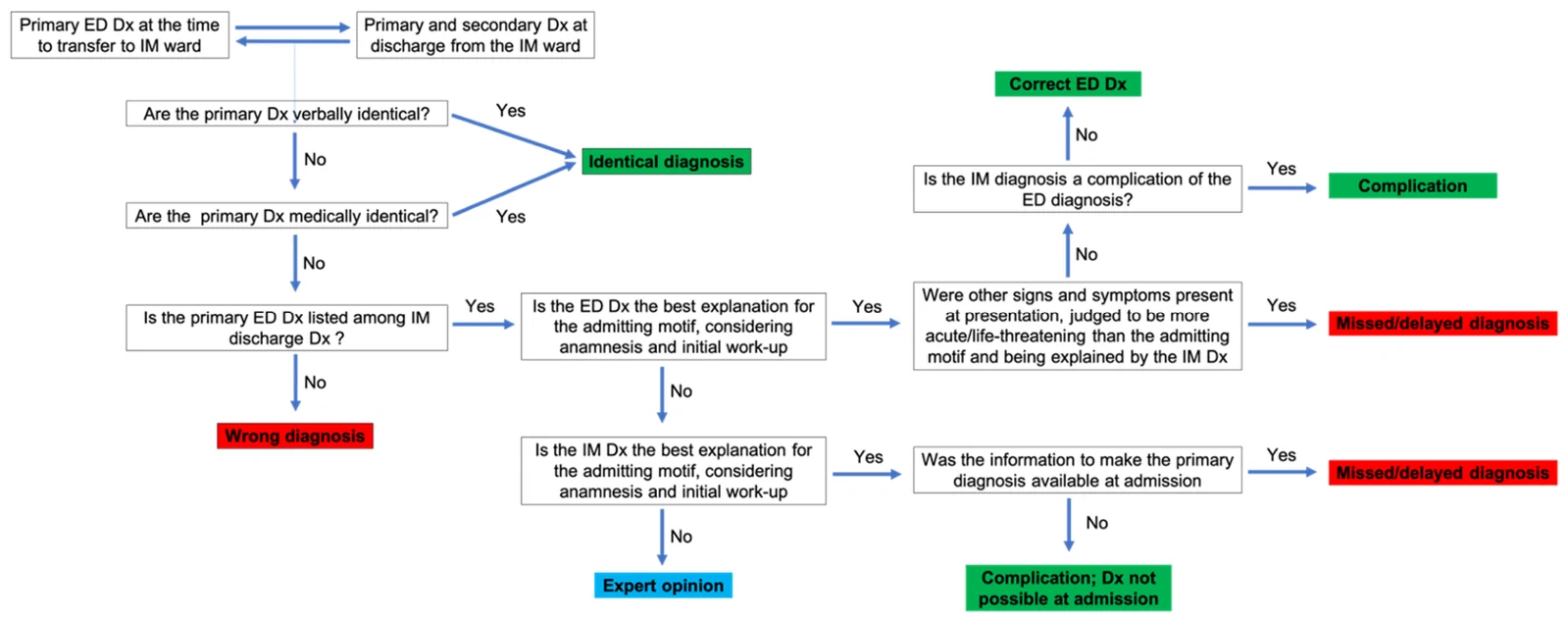
Algorithm to evaluate diagnostic discrepancies between emergency department and internal medicine ward diagnoses. ED: Emergency Department; IM: Internal Medicine; Dx: Diagnosis.

Diagnostic discrepancies were assessed using a predefined algorithm (Figure 1). The algorithm followed a hierarchical structure in which each step required a binary (yes/no) response, leading either to a definitive classification or to a subsequent level of analysis. The algorithm was developed based on existing models [4], clinical expertise, and the definition of diagnostic error described as a substantial discrepancy between the ED admittance diagnosis and the primary hospital discharge diagnosis [16]. The tool was designed to determine whether the ED diagnosis was correct (identical diagnoses, complications of hospital stay, diagnosis not reasonably identifiable at the time of ED assessment) or incorrect (wrong or missed/delayed). To improve consistency, the algorithm was initially tested and validated by an expert panel.

During the study, the principal investigator applied the algorithm to each case and was able to classify 381 of the 515 cases on their own (~74%). In case of uncertainty at any stage of the process, the case was reviewed by two senior physicians (“experts”) who used the algorithm in parallel; this occurred in 134 of the 515 cases (~26%). If a disagreement persisted between these two experts, a third expert with access to the full clinical record was consulted to reach a final consensus; this occurred in ~25% (33/134) of the cases previously reviewed by the two independent experts. None of the raters played any role in the clinical management of the cases evaluated to minimize bias. Patient anonymity was guaranteed by a study assistant who removed all identifying information prior to review.

### 2.5. Statistical Analysis

#### 2.5.1. Sample Size Rationale

The target sample size was set at 500 participants, following the statistical rationale recently established in a comparable Swiss clinical setting [4,20]. This estimate was derived from a power analysis using a significance level (alpha) of 0.05 and a statistical power of 85%. The calculation accounted for eight independent variables for the primary outcome (diagnostic discrepancy), an assumed effect size R of 0.2, and a 15% anticipated dropout rate. Given the similarities in this study’s objectives and hospital setting, this sample size was considered sufficient to ensure robust statistical power for our analysis.

#### 2.5.2. Missing Data and Model Robustness

Missing data were minimal. The primary outcome was complete for all 515 patients, and among the five predictors of the final model a single observation was missing (specialist involvement). The primary analysis was therefore performed on complete cases (*n* = 514), with MICE reported as a sensitivity analysis.

#### 2.5.3. Descriptive and Inferential Statistics

Descriptive statistics are presented as mean ± standard deviation (SD) for continuous variables or count and frequency (percentage) for categorical variables.

Group comparisons were performed using Welch’s *t*-test for continuous variables and chi-squared or Fisher’s exact tests for categorical variables, as appropriate.

#### 2.5.4. Regression Models

To evaluate associations between diagnostic discrepancy and clinical outcomes, several regression models were used.

Mortality and in-hospital transfer (binary outcomes) were analyzed using logistic generalized linear mixed-effects models (GLMMs) with diagnostic discrepancy as the main independent variable and physician ID included as a random intercept to account for clustering. Models were first fitted unadjusted, and then adjusted for potential confounders (age, sex, and triage category). These models estimate odds ratios with 95% confidence intervals.

Length of stay (LOS) was log-transformed to normalize residuals and analyzed using linear mixed-effects models with the same covariates and the same physician random intercept.

To identify factors associated with a change in diagnosis, we established a predictor dataset including patient-related, physician-related, and contextual variables. Each predictor was first assessed in a univariable logistic regression, with diagnostic discrepancy as the dependent variable.

Candidate variables were then entered into a stepwise selection procedure based on the Akaike Information Criterion (AIC) [21], run on a single imputed dataset (MICE). The selected model was subsequently modified in two respects: perceived diagnostic difficulty was treated as a numerical score from 1 to 5, and atypical presentation was excluded because its coefficients were not estimable (quasi-complete separation). No further selection procedure was applied after these modifications. The final model was fitted on complete cases, with multiple imputation (m = 5) reported as a sensitivity analysis.

The significance of predictors was assessed using *p*-values and 95% confidence intervals. Variables with *p* < 0.05 were considered statistically significant. Because we only tested for prior hypotheses, no correction for multiple testing was applied.

It should be noted that the study was not powered to detect an association with mortality as 32 deaths occurred overall, of which 5 occurred in the discrepancy group.

All analyses were performed using R version 4.5.1 (13 June 2025).

## 3. Results

### 3.1. Study Population

Out of 58,580 initial ED visits recorded during the study period, 3971 patients were admitted to the internal medicine ward. Of these, 515 patients, managed by 44 physicians, were ultimately included in the study. A diagnostic discrepancy was identified in 10.1% of patients (*n* = 52), while 89.9% of patients (*n* = 463) had no diagnostic discrepancy. Among all included patients, “missed/delayed diagnosis” and “wrong diagnosis” each accounted for 5.05% of the total cohort (*n* = 26). Inter-rater reliability between the two independent experts was substantial across the seven algorithm categories (Cohen’s kappa of 0.68). For the three-category classification that determines the study outcome (discrepancy, no discrepancy, expert review required) agreement was higher (Cohen’s kappa of 0.75).

The cohort had a mean age of 67.6 ± 17.9 years, with 41% (*n* = 212) female participants. Overall, 91% of patients had at least one known comorbidity. Cardiovascular disease was the most common comorbidity (71%), followed by pulmonary disease (28%), psychiatric disease (25%) and diabetes (23%). In total, 70% of patients fulfilled criteria for multimorbidity, defined as living with two or more chronic illnesses. Polypharmacy was present in 66% of patients. Of the 44 included physicians, 27% were male and 73% female, while their mean age was 27.66 ± 2.76 years. The number of included cases per physician was unevenly distributed, with a median of 3 cases per physician (range 1–77); the five largest contributors accounted for 51% of all included cases, and 15 physicians contributed a single case.

**Figure 2:**
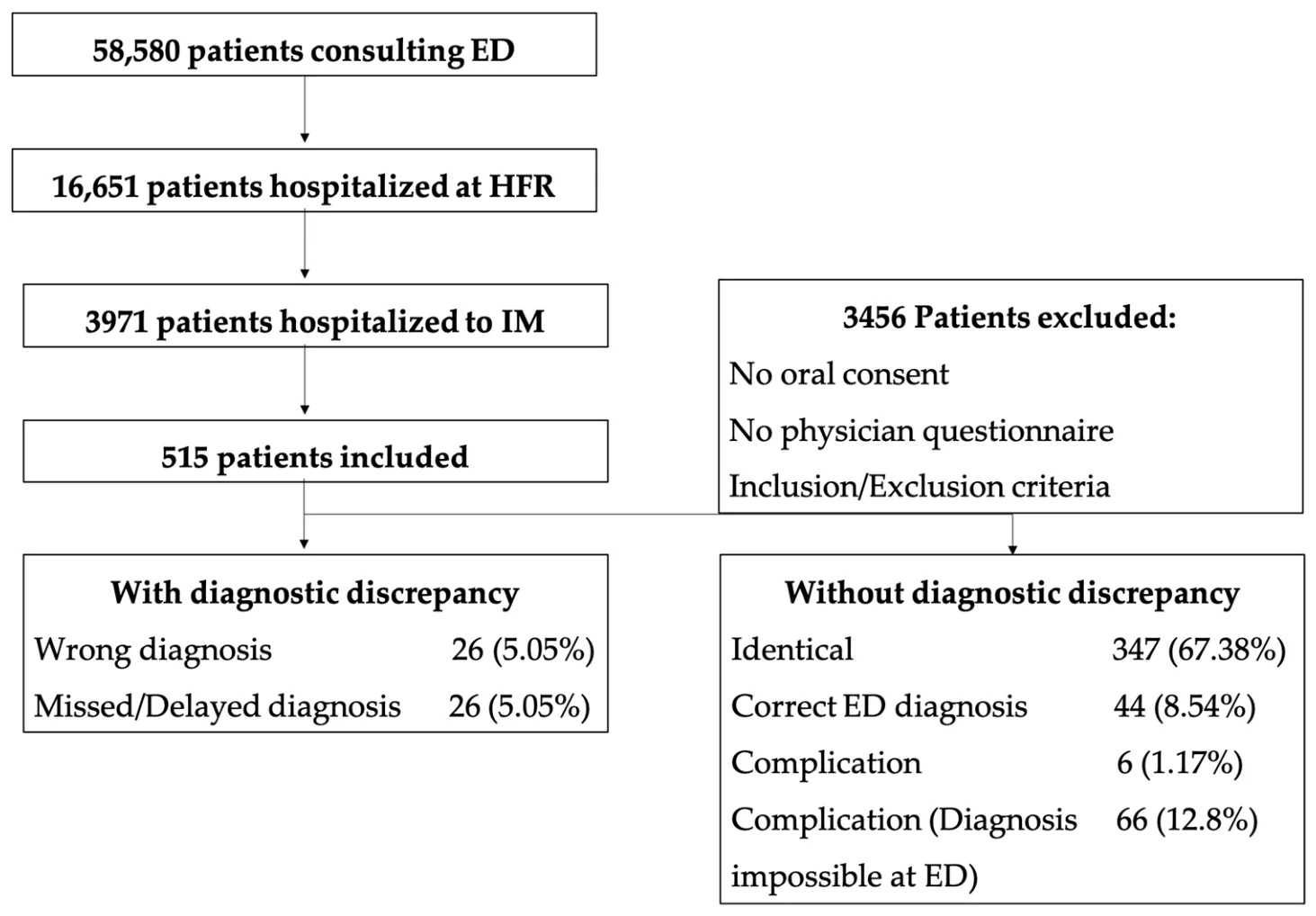
Study procedure.

The diagnoses involved in discrepancies were highly heterogeneous, spanning cardio-vascular, infectious, oncological, surgical, and neurological conditions, with no single diagnostic category or disease entity accounting for a predominant share of discrepancies (Figure 2).

### 3.2. Impact of Diagnostic Discrepancy on Clinical Outcomes

In the univariable analysis, patients with a diagnostic discrepancy had a mean length of stay of 9.3 ± 9.2 days compared to 6.9 ± 5.8 days for those without (*p* = 0.069). They were also more frequently transferred within the hospital (17% vs. 5.8%; *p* = 0.006) and showed a higher unadjusted mortality rate (9.6% vs. 5.8%; *p* = 0.4) (Table 1).

After adjusting for age, sex, and triage category using multivariable models, the presence of a diagnostic discrepancy remained independently associated with a 20% longer hospital stay (exp β = 1.20; 95% CI 1.00–1.45; *p* = 0.049) and significantly higher odds of in-hospital transfer (OR 3.35; 95% CI 1.47–7.66; *p* = 0.004) (Table 2). The estimate for mortality pointed in the same direction but did not reach statistical significance (OR 1.70; 95% CI 0.61–4.76; *p* = 0.30).

Beyond diagnostic discrepancies, older age emerged as a strong independent predictor of clinical outcomes in multivariable models. Specifically, each additional year of age was associated with 7% higher odds of in-hospital mortality (OR 1.07; 95% CI 1.03–1.11; *p* < 0.001). In addition, increasing age was consistently associated with a longer LOS (exp β = 1.01; 95% CI 1.01–1.01; *p* < 0.001).

In separate models including diagnostic discrepancy and comorbidity burden (Charlson Comorbidity Index), the association with in-hospital transfer persisted (OR 3.20; 95% CI 1.41–7.30; *p* = 0.006), whereas the association with length of stay was attenuated and no longer statistically significant (exp β 1.18; 95% CI 0.98–1.41; *p* = 0.076). The Charlson index was itself independently associated with both outcomes (transfer OR 1.11, *p* = 0.042; LOS exp β 1.06, *p* < 0.001).

### 3.3. Factors Predicting Diagnostic Discrepancy

#### 3.3.1. Univariable Analysis

In univariable analysis, being seen by a specialist in the ED was the only predictor significantly associated with lower odds of discrepancy (OR 0.35; 95% CI 0.12–0.82; *p* = 0.029). Other factors, including patient age, sex, comorbidities and multimorbidity, were not statistically significant predictors on their own.

#### 3.3.2. Multivariable Analysis

The final model was fitted on the complete cases (*n* = 514, 52 events), with multiple imputation (m = 5) reported as a sensitivity analysis.

The variable most strongly associated with lower odds of diagnostic discrepancy was specialist involvement in the ED (consultant from other specialties). Patients seen by a specialist had significantly lower odds of diagnostic discrepancy compared to those seen by residents alone (OR 0.30; 95% CI 0.10–0.72; *p* = 0.015).

Conversely, diagnostic difficulty, as self-reported by the physician, was independently associated with diagnostic discrepancy. Each one-point increase in physician-reported diagnostic difficulty was associated with higher odds of diagnostic discrepancy (OR 1.42; 95% CI 1.07–1.90; *p* = 0.016). Other potential predictors, such as comorbidities and physician experience, were not statistically significant in the final adjusted model (Table 3). Estimates were stable across sensitivity analyses.

## 4. Discussion

### 4.1. Main Findings

This prospective observational study provides a detailed analysis of diagnostic performance in a Swiss ED. Our main findings are threefold: (1) diagnostic discrepancies occurred in 10.1% of patients admitted to internal medicine; (2) these discrepancies were associated with higher rates of in-hospital transfers and, less robustly, with longer hospital stays; and (3) early involvement of a specialist in the ED (other than a specialist in emergency medicine) was associated with substantially lower odds of diagnostic discrepancy (approximately 70%).

### 4.2. Interpretation of Discrepancy Rates

Our observed discrepancy rate of 10.1% falls within the range of 5% to 15% reported in comparable international studies [1,4]. Although our study was not powered or designed to enable direct comparison between the two institutions, another prospective study from the University Hospital in Bern, examining diagnostic change between the ED and IM, reported a rate of 12.3% [4]. Beyond the setting, our study differs in that it was conducted in a cantonal non-university hospital with a less experienced physician population, applied a newly developed hierarchical algorithm, and examined specialist involvement in the ED as an organizational factor.

Furthermore, differences in the conceptualization and operationalization of diagnostic discrepancy must be considered. We considered an ED diagnosis to be accurate if it accounted for the patient’s presenting complaint and did not overlook any critical warning signs, even if the discharge diagnosis ultimately differed [4], whereas others emphasized outcome-based measures.

Although diagnostic discrepancy and diagnostic error are distinct concepts and should not be interpreted interchangeably, our findings are broadly consistent with literature on diagnostic errors. A 2022 systematic review estimated a pooled diagnostic error rate of 5.7%, based on administrative data and literature-derived rates. However, it also reported a wide variability in error rates depending on disease type, ranging from 1.5% for myocardial infarction to 56% for spinal abscess, highlighting the complexity of measuring diagnostic accuracy [1].

### 4.3. Associations with Clinical Outcomes and Organization

Diagnostic discrepancies were associated with longer hospital stays and a greater likelihood of in-hospital transfer. In our study, the presence of a diagnostic discrepancy was associated with a 20% longer hospital stay and 3.35-fold-higher odds of being transferred within the hospital.

These findings are consistent with prior studies, which have demonstrated that patients with diagnostic discrepancies experience increased lengths of stay ranging from an additional 0.76 to 3.4 days compared to those without such discrepancies [3,4,22]. Additionally, increased rates of in-hospital transfer among patients with diagnostic error have been well-documented [23].

However, it remains unclear whether the diagnostic discrepancies identified in this study directly contribute to the increased LOS and in-hospital transfers, or whether patients with inherently more complex diseases, who are already at higher risk of poor outcomes, are also more likely to have a discrepant diagnosis.

We found a 7% rise in the odds of death per additional year of age, and while diagnostic discrepancies themselves were not directly associated with mortality, the strong age effect suggests that older patients’ worse outcomes are likely driven more by their underlying vulnerability than by diagnostic discrepancy per se.

Patients with a diagnostic discrepancy exhibited 1.70 times higher odds of mortality compared to those without a discrepancy, but this association did not reach statistical significance. This lack of statistical significance may be attributable to the relatively small number of deaths. However, in the literature, diagnostic error has been associated with an absolute increase in mortality ranging from 0.2% to 4.8% [1,4].

### 4.4. Factors Associated with Diagnostic Discrepancy

Diagnostic difficulty reported by physicians was linked to higher odds of discrepancy, although this association should be regarded as exploratory given that perceived diagnostic difficulty did not reach significance in univariable analysis or under a binary coding of the scale. Nevertheless, this finding is aligned with results from a similar study, which reported that physicians’ perception of an atypical clinical presentation was a significant predictor of diagnostic change [4]. These findings underline the value of physicians’ intuition or “gut feeling”, and the continuing role of human clinical judgment.

Specialist involvement in the ED was associated with a significantly lower likelihood of diagnostic discrepancy, corresponding to 70% lower odds of diagnostic change. However, this association should not be interpreted as evidence that specialist involvement causally reduces discrepancies. Indeed, such involvement was not randomly assigned but depended on clinical judgement, and the criteria for requesting a specialist may vary between physicians, creating a risk of indication bias.

In our institution, residents discussed each case with a supervisor from the ED who was board-certified in IM or EM. Further specialist involvement (labeled as “seen by specialist”), when deemed necessary by the supervisor, was sought to support further diagnostic workup or to provide immediate treatment.

To our knowledge, this is the first prospective study to demonstrate an independent association between specialist involvement and lower odds of diagnostic discrepancy. We found no previous studies directly examining this association; however, our findings are consistent with a retrospective study reporting that reported that diagnostic discrepancies occurred more frequently when patients were initially assessed by interns than by residents [22]. Physicians in specialist rotations are typically more advanced in their specific field, and in our institution each case was discussed with a board-certified specialist who possesses extensive expertise.

The lower odds observed may also relate to specialists’ tendency to initiate more thorough diagnostic workups early on. Previous studies have identified early and detailed history-taking and timely investigations as critical determinants of diagnostic accuracy [22,23].

Another possible explanation is that expert opinions are less likely to be challenged during the hospital stay, resulting in fewer changes in diagnosis. Specialists are typically consulted only in cases of high diagnostic uncertainty or when the condition clearly falls within their field of expertise.

Furthermore, specialists are also consulted when the diagnosis is relatively straightforward but their expertise is required to initiate timely treatment. For example, the diagnostic accuracy of ST-elevation myocardial infarction (STEMI) or acute stroke is generally high, yet cardiologists and neurologists are involved early in the ED to facilitate definitive management. Consequently, specialist involvement reflects not only diagnostic uncertainty but also established clinical pathways requiring specialty-specific treatment.

The primary role of emergency physicians is to identify and manage acute medical conditions and to refer patients to the appropriate specialist when necessary. As such, not all patients are evaluated by specialists; referrals generally occur only after common conditions have been excluded and more specific expertise is required. Furthermore, the early initiation of more extensive diagnostic workups by experts may put patients at risk for overdiagnosis.

The relationship between perceived diagnostic difficulty and specialist involvement remains complex. It is unclear whether physicians call a specialist because the case clearly falls within that specialty’s domain, implying that the problem itself is straightforward, or because diagnostic uncertainty is high. Further studies are needed to address this question.

Interestingly, other commonly proposed predictors such as comorbidities, clinician experience level, and ED crowding, were not statistically significant in our analysis.

### 4.5. Strengths and Limitations

The primary strength of this study is its prospective observational design, which captures the reality of clinical decision-making more accurately than retrospective chart reviews. Additionally, we employed rigorous statistical methods, using GLMMs to account for confounding factors and the clustering effect of physicians.

However, the interpretation of our findings is limited by several methodological constraints.

First, the number of diagnostic discrepancies was relatively small (*n* = 52), which restricts model stability and increases the likelihood of imprecise or unstable estimates. The final model comprises five predictors for 52 events, corresponding to approximately ten events per variable. This does not eliminate the risk of overfitting, and the analysis of factors associated with diagnostic discrepancy should be regarded as exploratory.

Second, only 515 of the 3971 patients admitted from ED to an internal medicine department during the study period were included. Non-inclusion resulted from the absence of consent, from non-completion of the questionnaire, or from both, and inclusion consequently depended on patient-level as well as physician-level factors. As neither the reason for non-inclusion nor data on patients not included in the study were collected, we were unable to compare the two groups and cannot determine whether the included sample is representative of the overall population. Cases deemed simple, or those managed during periods of low activity, may have been included preferentially, and it is impossible to determine the direction of any resulting bias. The observed discrepancy rate of 10.1% should therefore be interpreted as applying to the sampled population rather than to all ED-to-IM admissions.

Third, although patients admitted for palliative care were excluded, several individuals transitioned to palliative status during hospitalization and were not excluded, which may have influenced outcome classification.

Fourth, the true rate of diagnostic discrepancy may be either overestimated or underestimated. It may be overestimated because we used the internal medicine discharge diagnosis as the reference standard; although it reflects the diagnosis under which the patient improved sufficiently to be discharged, it does not guarantee diagnostic correctness. Moreover, cases in which symptoms temporarily resolved with supportive care, despite incomplete or inaccurate diagnosis, may have been misclassified as having no discrepancy. Because the primary outcome is defined relative to this imperfect reference standard, our findings should be interpreted with caution, and no causal claims about the correctness of either diagnosis can be drawn. In addition, the principal investigator was not blinded to clinical outcomes, as the discharge letters used for classification may report length of stay, in-hospital transfer or death. A hindsight bias in the classification of diagnostic discrepancies can therefore not be excluded, and neither its magnitude nor its direction can be determined.

Conversely, the discrepancy rate may be underestimated compared with other studies, as our definition focused on diagnostic discrepancy within the ED and prioritized explanations for the chief complaint, provided that no red flags were missed. This narrower definition may classify some cases as “accurate” that would be considered discrepant under broader criteria used in other settings.

### 4.6. Future Research

Our study identified an association between diagnostic discrepancies and adverse outcomes, but further multicenter prospective trials are needed to validate these findings, improve their generalizability and assess causality. Literature indicates that the vast majority of diagnostic errors stem from failures in clinical reasoning and decision-making rather than lack of knowledge [1,17]. Interventional studies should evaluate whether structured diagnostic support strategies, such as early specialist consultation or diagnostic escalation based on physicians’ perceived diagnostic difficulty, can reduce diagnostic discrepancies and improve patient outcomes. Further research is also needed to better understand the mechanisms underlying the association between specialist involvement and lower rates of diagnostic discrepancy, including the risks associated with over-diagnosing.

## 5. Conclusions

Diagnostic discrepancies are a frequent problem in the ED, affecting one in ten patients admitted to internal medicine. They were independently associated with increased in-hospital transfers, and less robustly with prolonged hospital stays. Specialist involvement was independently associated with lower odds of diagnostic discrepancy, although this finding should be interpreted with caution given the observational design and the possibility of indication bias, and without excluding possible risks of over-diagnosis. Physicians’ subjective perception of diagnostic difficulty may also represent a valuable early warning sign, warranting further prospective evaluation.

## Figures and Tables

**Table 1:** Differences between patients with and without diagnostic discrepancy.

Characteristic	Overall N = 515 ^1^	No Discrepancy N = 463 ^1^	Discrepancy N = 52 ^1^	*p*-Value ^2^
Age (years)	67.6 (17.9) (17.0–98.0)	67.3 (17.9) (17.0–98.0)	69.6 (17.9) (17.0–92.0)	0.4
Sex				0.3
Female	212 (41%)	187 (40%)	25 (48%)	
Male	303 (59%)	276 (60%)	27 (52%)	
Triage category ^3^				0.5
U1	78 (15%)	71 (15%)	7 (13%)	
U2	249 (48%)	221 (48%)	28 (54%)	
U3	95 (18%)	89 (19%)	6 (12%)	
U3 ^4^	93 (18%)	82 (18%)	11 (21%)	
Comorbidity	468 (91%)	418 (90%)	50 (96%)	0.2
Polypharmacy	338 (66%)	298 (64%)	40 (77%)	0.071
Frailty	4.4 (1.9) (1.0–9.0)	4.4 (1.9) (1.0–9.0)	4.7 (1.9) (2.0–9.0)	0.3
LOS total (days)	7.2 (6.2) (0.0–55.0)	6.9 (5.8) (0.0–55.0)	9.3 (9.2) (1.0–53.0)	0.069
Mortality	32 (6.2%)	27 (5.8%)	5 (9.6%)	0.4
LOS in ED (min)	451.6 (211.2) (0.0–1453.0)	450.7 (208.9) (0.0–1453.0)	459.7 (232.7) (0.0–1440.0)	0.8
In-hospital transfer	36 (7.0%)	27 (5.8%)	9 (17%)	0.006
Transferred to other hospital	26 (5.0%)	23 (5.0%)	3 (5.8%)	0.7
Discharge orientation				0.6
Home	370 (72%)	335 (72%)	35 (67%)	
Other	39 (7.6%)	33 (7.1%)	6 (12%)	
Retirement Home	17 (3.3%)	15 (3.2%)	2 (3.8%)	
Transfer to other hospital (acute care)	17 (3.3%)	16 (3.5%)	1 (1.9%)	
Transfer to other specialty	17 (3.3%)	14 (3.0%)	3 (5.8%)	
Transfer to rehabilitation	55 (11%)	50 (11%)	5 (9.6%)	

^1^ Mean (SD) (Min–Max); *n* (%); ^2^ Welch Two Sample *t*-test; Fisher’s exact test; Pearson’s Chi-squared test; ^3^ U3 triage criteria with additional vulnerability criteria (e.g., elderly patients, severe pain, oncological disease, etc.); ^4^ Echelle Suisse de Tri (EST).

**Table 2:** Association between diagnostic discrepancy and clinical outcomes (Multivariable Models adjusted for age, sex, and triage).

Predictor	Mortality ^¥^	In-Hospital Transfer ^¥^	Length of Hospital Stay ^§^
Diagnostic discrepancy (yes)	1.70 (0.61–4.76); *p* = 0.30	3.35 (1.47–7.66); *p* = 0.004	1.20 (1.00–1.45); *p* = 0.049
Age (per year)	1.07 (1.03–1.11); *p* < 0.001	1.02 (0.99–1.04); *p* = 0.2	1.01 (1.01–1.01); *p* = <0.001
Sex (male vs. female)	1.59 (0.73–3.44); *p* = 0.20	1.21 (0.6–2.46); *p* = 0.60	1.04 (0.93–1.17); *p* = 0.5
Triage category (vs. U1)			
U2	0.54 (0.21–1.36); *p* = 0.2	0.72 (0.29–1.84); *p* = 0.5	1.06 (0.90–1.25); *p* = 0.50
U3	0.65 (0.18–2.35); *p* = 0.50	0.70 (0.20–2.38); *p* = 0.6	1.08 (0.88–1.31); *p* = 0.50
U3 ^1^	0.48 (0.15–1.50); *p* = 0.20	0.73 (0.24–2.23); *p* = 0.6	1.09 (0.90–1.32); *p* = 0.4

All models were adjusted for diagnostic discrepancy, age, sex, and triage category, with physician ID included as a random intercept to account for clustering of patients seen by the same doctor. ^¥^ Logistic Generalized Linear Mixed Model: [OR (95% CI); *p*-value]; ^§^ Linear Mixed Model, log scale [exp β (95% CI); *p*-value]. ^1^ U3 triage criteria with additional vulnerability criteria (e.g., elderly patients, severe pain, oncological disease, etc.).

**Table 3:** Predictors of diagnostic discrepancy. Final complete-case model, logistic regression.

Predictor	OR	95% CI	*p*-Value
Seen by specialist (Yes vs. No)	0.30	0.10–0.72	0.015
Perceived diagnostic difficulty (per 1-point increase)	1.42	1.07–1.90	0.016
Comorbidities (Yes vs. No)	3.10	0.89–19.7	0.13
Male sex (vs. Female)	0.64	0.35–1.16	0.14
Physician EM experience (per month)	1.02	0.99–1.05	0.2

## Data Availability

Data are available to researchers who comply with the Swiss regulations for working with anonymized health care data upon reasonable request from the corresponding author.

## Supplementary Materials

The following supporting information can be downloaded at https://www.mdpi.com/article/10.3390/jcm15187177/s1. Supplemental Data S1: Physician Questionnaire (1st), Supplemental Data S2: Physician Questionnaire (2nd).

## Abbreviations

The following abbreviations are used in this manuscript:

AIC	Akaike Information Criterion
ED	Emergency Department
GLMM	Generalized linear mixed-effects model
HFR	Fribourg Cantonal Hospital
ICU	Intensive care unit
IM	Internal Medicine
LOS	Length of stay
MICE	Multiple Imputation by Chained Equations
OECD	Organisation for Economic Co-operation and Development
EM	Emergency Medicine

## References

[1] Newman-Toker D.E., Peterson S.M., Badihian S., Hassoon A., Nassery N., Parizadeh D., Wilson L.M., Jia Y., Omron R., Tharmarajah S. (2022). Diagnostic Errors in the Emergency Department: A Systematic Review.

[2] Newman-Toker D.E., Nassery N., Schaffer A.C., Yu-Moe C.W., Clemens G.D., Wang Z., Zhu Y., Saber Tehrani A.S., Fanai M., Hassoon A. (2024). Burden of serious harms from diagnostic error in the USA. BMJ Qual. Saf..

[3] Johnson T., McNutt R., Odwazny R., Patel D., Baker S. (2009). Discrepancy between admission and discharge diagnoses as a predictor of hospital length of stay. J. Hosp. Med..

[4] Hautz W.E., Kämmer J.E., Hautz S.C., Sauter T.C., Zwaan L., Exadaktylos A.K., Birrenbach T., Maier V., Müller M., Schauber S.K. (2019). Diagnostic error increases mortality and length of hospital stay in patients presenting through the emergency room. Scand. J. Trauma Resusc. Emerg. Med..

[5] Singh H., Giardina T.D., Meyer A.N.D., Forjuoh S.N., Reis M.D., Thomas E.J. (2013). Types and origins of diagnostic errors in primary care settings. JAMA Intern. Med..

[6] Schaffer A.C., Jena A.B., Seabury S.A., Singh H., Chalasani V., Kachalia A. (2017). Rates and Characteristics of Paid Malpractice Claims Among US Physicians by Specialty, 1992–2014. JAMA Intern. Med..

[7] Slawomirski L., Kelly D., de Bienassis K., Kallas K.A., Klazinga N. (2025). The Economics of Diagnostic Safety.

[8] Berner E.S., Graber M.L. (2008). Overconfidence as a cause of diagnostic error in medicine. Am. J. Med..

[9] Peng A., Rohacek M., Ackermann S., Ilsemann-Kakaroumis J., Ghanim L., Messmer A., Misch F., Nickel C., Bingisser R. (2015). The proportion of correct diagnoses is low in emergency patients with nonspecific complaints presenting to the emergency department. Swiss Med. Wkly..

[10] Hussain F., Cooper A., Carson-Stevens A., Donaldson L., Hibbert P., Hughes T., Edwards A. (2019). Diagnostic error in the emergency department: Learning from national patient safety incident report analysis. BMC Emerg. Med..

[11] Newman-Toker D.E., Makary M.A. (2013). Measuring Diagnostic Errors in Primary Care: The First Step on a Path Forward Comment on “Types and Origins of Diagnostic Errors in Primary Care Settings”. JAMA Intern. Med..

[12] Mattsson B., Ertman D., Exadaktylos A.K., Martinolli L., Hautz W.E. (2018). Now you see me: A pragmatic cohort study comparing first and final radiological diagnoses in the emergency department. BMJ Open.

[13] Andrews L.B., Stocking C., Krizek T., Gottlieb L., Krizek C., Vargish T., Siegler M. (1997). An alternative strategy for studying adverse events in medical care. Lancet.

[14] Battle R.M., Pathak D., Humble C.G., Key C.R., Vanatta P.R., Hill R.B., Anderson R.E. (1987). Factors influencing discrepancies between premortem and postmortem diagnoses. JAMA.

[15] Singh H., Graber M. (2010). Reducing Diagnostic Error through Medical Home-Based Primary Care Reform. JAMA.

[16] Graber M.L., Franklin N., Gordon R. (2005). Diagnostic error in internal medicine. Arch. Intern. Med..

[17] Hautz W.E. (2018). When I say… diagnostic error. Med. Educ..

[18] Zwaan L., Singh H. (2015). The challenges in defining and measuring diagnostic error. Diagnosis.

[19] Swissethics (2019). Distinction Between Research and Quality Assurance.

[20] Hautz S.C., Schuler L., Kämmer J.E., Schauber S.K., Ricklin M.E., Sauter T.C., Maier V., Birrenbach T., Exadaktylos A., Hautz W.E. (2016). Factors predicting a change in diagnosis in patients hospitalised through the emergency room: A prospective observational study. BMJ Open.

[21] Akaike H., Parzen E., Tanabe K., Kitagawa G. (1998). Information Theory and an Extension of the Maximum Likelihood Principle. Selected Papers of Hirotugu Akaike Springer Series in Statistics.

[22] Fatima S., Shamim S., Butt A.S., Awan S., Riffat S., Tariq M. (2021). The discrepancy between admission and discharge diagnoses: Underlying factors and potential clinical outcomes in a low socioeconomic country. PLoS ONE.

[23] Chiu H., Chan K., Chung C., Ma K., Au K. (2003). A Comparison of Emergency Department Admission Diagnoses and Discharge Diagnoses: Retrospective Study. Hong Kong J. Emerg. Med..

