# Supplementary material for: Diagnostic Discrepancies Between Emergency Department Assessment and Internal Medicine Discharge Diagnosis: A Prospective Observational Study in a Swiss Tertiary Hospital
Source: J Clin Med. 2026 Sep 15;15(18):7177. doi: 10.3390/jcm15187177 (PMC13607261; doi:10.3390/jcm15187177)
Supplement: Supplementary file 1 [file jcm-15-07177-s001.zip › Supplemental Data S1.pdf]

Supplemental Data S1- Diagnostic discrepancies between emergency department as-sessment and hospital discharge diagnosis: a prospective ob-servational study in Switzerland

Theresa Ackfeld [1,2,*], Youcef Guechi [1,2,3], Joseph Schwab [4], Thomas Castelain [1,2], Ludovic Galofaro [1,2], Cynthia Gay [1], Sébastien Pugnale [1], Thomas Schmutz [5], Wolf E. Hautz [6] and Vincent Ribordy [1,2]

Supplemental Data S1: Physician Questionnaire (1st)

Physician Questionnaire (1st)

Takes 3 minutes to answer

Date________________

Physicians acronym (consisting of the first two letters of your mothers first name, the first two letters of your fathers first name, day of your own birthday. For example LEHA05 for Lena and Hans, own birthday on May 5th.):____________________________

Thank you for participating in the “Diagnostic performance study”. Please return the questionnaire into the folder “Diagnostic change ED” at conference room “Gastlosen”. You need to fill out this questionnaire only at the beginning of the study. For each patient please refer to the Physician Questionnaire (2nd)

Your Gender?
☐ Female ☐ Male

Your Age? __________

What languages do you speak fluently
☐ German ☐ French ☐ other_________
What is your mother tongue: ☐ Swiss-german (Dialect) ☐ German (High-German) ☐ French ☐ other_________

Year of approbation__________________
Months of experience in internal medicine ________________
Months of experience in emergency medicine _______________
Months of experience in total _________________

What is your current function in the ER?
☐ Consultant (médecin adjoint)
☐ Attending (CDC)
☐ Intern (médecin assistant)
☐ Medical Intern (stagiare)

Which specialty training hired you?
☐ Surgical
☐ Medical (internal medicine)
☐ Emergency Medicine

What is your planned specialty
☐ Surgical
☐ Medical (internal medicine)
☐ General practice
☐ Emergency Medicine
☐ Specialization (Cardiology, angiology, nephrology, etc. please precise):________________
☐ Not decided yet

Do you already have a specialty degree?
☐ Yes
☐ No

Thank you for your support. With questions or suggestions please contact Theresa Ackfeld (Theresa.ackfeld@h-fr.ch, 62457)
