# Supplementary material for: Diagnostic Discrepancies Between Emergency Department Assessment and Internal Medicine Discharge Diagnosis: A Prospective Observational Study in a Swiss Tertiary Hospital
Source: J Clin Med. 2026 Sep 15;15(18):7177. doi: 10.3390/jcm15187177 (PMC13607261; doi:10.3390/jcm15187177)
Supplement: Supplementary file 1 [file jcm-15-07177-s001.zip › Supplemental Data S2.pdf]

Supplemental Data S2- Diagnostic discrepancies between emergency department as-sessment and hospital discharge diagnosis: a prospective ob-servational study in Switzerland

Theresa Ackfeld [1,2,*], Youcef Guechi [1,2,3], Joseph Schwab [4], Thomas Castelain [1,2], Ludovic Galofaro [1,2], Cynthia Gay [1], Sébastien Pugnale [1], Thomas Schmutz [5], Wolf E. Hautz [6] and Vincent Ribordy [1,2]

Supplemental Data S2: Physician Questionnaire (2nd)

Takes 3-5 minutes to fill out
This questionnaire has to be filled out **for every patient hospitalized** to the internal medicine ward

Physicians acronym:______________________________
Name and date of birth of the patient____
The following questions refer to <u>this</u> patient

1) Have you seen this patient from the beginning to the end? Yes   No
2) Only if you choose No: How many hand-overs have there been: 1 2 3 >3
3) Was this patient seen by a CDC? Yes   No
4) Has this patient been seen by an internal medicine assistant (In the ED)? Yes   No
5) Has this patient been seen by an internal medicine CDC (In the ED) ? Yes   No
6) Did internal medicine ask for further investigations?
    Yes   No
   If yes, which ones: lab work imagery studies expert opinion
   ____
7) Did internal medicine change the diagnosis? Yes   No
8) Has this patient been seen by a specialist (In the ED)? Yes   No

The following statements are correct:

9) I am confident, that the diagnosis I assigned is correct?
    strongly agree agree neutral disagree strongly disagree
10) If the diagnosis was difficult to establish did you communicate this⑤
   ⑤to the patient? Yes No
   ⑤ to your supervisor? Yes No
11) Did you and your primary supervisor agree on the diagnosis? Yes No
12) Making the diagnosis was difficult
    strongly agree agree neutral disagree strongly disagree
13) The clinical presentation was atypical for the assumed diagnosis
    strongly agree agree neutral disagree strongly disagree
14) I used medical literature during the diagnostic process (uptodate, guidelines, amboss⑤) ?
    strongly agree agree neutral disagree strongly disagree
15) I used internal guidelines during the diagnostic process (HFR STEMI,⑤)
    strongly agree agree neutral disagree strongly disagree
16) I am used to the assumed diagnosis
    strongly agree agree neutral disagree strongly disagree
17) The key element in making the diagnosis was: history imaging laboratory discussion with my supervisor
18) The work load (imposed by all your patients) was high when I made the diagnosis
    strongly agree agree neutral disagree strongly disagree
19) I was tired when making the diagnosis
    strongly agree agree neutral disagree strongly disagree
20) I was able to communicate easily with the patient
    strongly agree (fluently) agree (operationally) neutral disagree (limited) strongly disagree (not at all)
21) Time of hand-over to the ward: ____________
22) Oral consentement obtained by the patient and utilization of data accepted by the patient ? Yes No
23) Workload in the ED green yellow red black

Please return the questionnaire into the folder “Diagnostic change ED” at the conference room “Gastlosen”. Thank you for your cooperation. If you have any questions or suggestions : Theresa.ackfeld@h-fr.ch , 62457
